# Reproducibility of quantitative (*R*)-[^11^C]verapamil studies

**DOI:** 10.1186/2191-219X-2-1

**Published:** 2012-01-17

**Authors:** Daniëlle ME van Assema, Mark Lubberink, Ronald Boellaard, Robert C Schuit, Albert D Windhorst, Philip Scheltens, Bart NM van Berckel, Adriaan A Lammertsma

**Affiliations:** 1 Department of Neurology & Alzheimer Center, PK-1Z035, VU University Medical Center, P.O. Box 7057, Amsterdam 1007 MB, The Netherlands; 2 PET Centre, Uppsala University Hospital, Uppsala 751 85, Sweden; 3 Department of Nuclear Medicine & PET Research, VU University Medical Center, PO Box 7057, Amsterdam 1007 MB, The Netherlands

**Keywords:** Positron emission tomography, P-glycoprotein, reproducibility, (*R*)-[^11^C]verapamil

## Abstract

**Background:**

P-glycoprotein [Pgp] dysfunction may be involved in neurodegenerative diseases, such as Alzheimer's disease, and in drug resistant epilepsy. Positron emission tomography using the Pgp substrate tracer (*R*)-[^11^C]verapamil enables *in vivo *quantification of Pgp function at the human blood-brain barrier. Knowledge of test-retest variability is important for assessing changes over time or after treatment with disease-modifying drugs. The purpose of this study was to assess reproducibility of several tracer kinetic models used for analysis of (*R*)-[^11^C]verapamil data.

**Methods:**

Dynamic (*R*)-[^11^C]verapamil scans with arterial sampling were performed twice on the same day in 13 healthy controls. Data were reconstructed using both filtered back projection [FBP] and partial volume corrected ordered subset expectation maximization [PVC OSEM]. All data were analysed using single-tissue and two-tissue compartment models. Global and regional test-retest variability was determined for various outcome measures.

**Results:**

Analysis using the Akaike information criterion showed that a constrained two-tissue compartment model provided the best fits to the data. Global test-retest variability of the volume of distribution was comparable for single-tissue (6%) and constrained two-tissue (9%) compartment models. Using a single-tissue compartment model covering the first 10 min of data yielded acceptable global test-retest variability (9%) for the outcome measure *K*_1_. Test-retest variability of binding potential derived from the constrained two-tissue compartment model was less robust, but still acceptable (22%). Test-retest variability was comparable for PVC OSEM and FBP reconstructed data.

**Conclusion:**

The model of choice for analysing (*R*)-[^11^C]verapamil data is a constrained two-tissue compartment model.

## Background

P-glycoprotein [Pgp] is considered to be the most important efflux transporter at the human blood-brain barrier [BBB] because of its high expression and its ability to transport a wide range of substrates from the brain into the circulation and cerebrospinal fluid. Pgp plays an important role in protecting the brain from endogenous and exogenous toxic substances by removing them before they reach the parenchyma [1-5]. It has been hypothesised that decreased Pgp function and/or expression at the BBB are involved in several neurological disorders, such as Creutzfeldt-Jakob disease, Parkinson's disease and Alzheimer's disease [AD] [6-9]. On the other hand, increased Pgp function may be involved in drug-resistant epilepsy [10].

Over the past years, several positron emission tomography [PET] tracers have been developed for quantifying Pgp function *in vivo*. Of these, (racemic) [^11^C]verapamil, (*R*)-[^11^C]verapamil and [^11^C]-*N*-desmethyl-loperamide have been used in humans [8,11-15]. Both (*R*) and (*S*) enantiomers of verapamil are substrates for Pgp, but (*R*)-[^11^C]verapamil is the preferred isomer for quantification of Pgp function as it is metabolised less than (*S*)-[^11^C]verapamil [16,17]. (*R*)-[^11^C]verapamil has been widely used both in healthy controls without [12,18-20] and with modulation of Pgp function [21,22] and in neurological diseases such as epilepsy [10], Parkinson's disease [11] and AD [9].

Several tracer kinetic models for quantification of (*R*)-[^11^C]verapamil data have been reported [19,23] with the standard single-tissue compartment model [1T2k] being used most frequently. An alternative approach is to apply the single-tissue compartment model only to the first 10 min after injection (1T2k^10^) in order to minimise effects of radiolabelled metabolites potentially crossing the BBB [23]. Other studies, however, have shown that a two-tissue compartment model [2T4k] provides good fits to the data, and a study using spectral analysis as well as studies in which Pgp was blocked pharmacologically suggests that indeed two compartments can be identified [9,21,23]. An important characteristic of a tracer kinetic model is its test-retest [TRT] variability. This not only determines group sizes in cross-sectional studies, but is also particularly important in longitudinal studies designed to assess changes over time or after treatment with disease-modifying drugs. To date, only one study has reported on TRT variability of (*R*)-[^11^C]verapamil data [19]. This study, however, did not include all tracer kinetic models mentioned above, and TRT variability was only reported for a whole brain region of interest [ROI]. Clearly, information about regional TRT variability is important in order to interpret changes in Pgp function in smaller anatomical structures. Therefore, the main aim of this study was to assess regional TRT variability of (*R*)-[^11^C]verapamil PET data for several tracer kinetic models. In addition, effects of correcting for partial volume effects on TRT variability were assessed.

## Materials and methods

### Subjects

Thirteen healthy controls, six males and seven females, were included (mean age 40 years, range 21 to 63 years). A subset of these data has been published previously as a part of the model development for (*R*)-[^11^C]verapamil [19]. Subjects were recruited through advertisements in newspapers and by means of flyers. All subjects were screened extensively for somatic and neurological disorders and had to fulfil research diagnostic criteria for having never been mentally ill. Screening procedures included medical history, physical and neurological examinations, screening laboratory tests of blood and urine, and brain magnetic resonance imaging [MRI] which was evaluated by a neuroradiologist. Subjects were not included if there was use of drugs of abuse or use of medication known to interfere with Pgp function [24,25]. Additional exclusion criteria were history of major neurological or psychiatric illness and clinically significant abnormalities of laboratory tests or MRI scan. Written informed consent was obtained from all subjects after a complete written and verbal description of the study. The study was approved by the Medical Ethics Review Committee of the VU University Medical Center.

### MRI

Six subjects underwent a structural MRI scan using a 1.0 T Magnetom Impact scanner (Siemens Medical Solutions, Erlangen, Germany) and seven subjects using a 1.5 T Sonata scanner (Siemens Medical Solutions, Erlangen, Germany). The scanning protocol on both scanners included an identical coronal T1-weighted 3-D magnetization-prepared rapid acquisition gradient-echo sequence (slice thickness = 1.5 mm; 160 slices; matrix size = 256 × 256; voxel size = 1 × 1 × 1.5 mm; echo time = 3.97 ms; repetition time = 2.70 ms; inversion time = 950 ms; flip angle = 8°). The MRI scan was used for co-registration and for ROI definition.

### PET data acquisition

All subjects underwent two identical PET scans on the same day. Scans were performed on an ECAT EXACT HR+ scanner (Siemens/CTI, Knoxville, USA), equipped with a neuro-insert to reduce the contribution of scattered photons from outside the field of view of the scanner. This scanner enables acquisition of 63 transaxial planes over a 15.5-cm axial field of view, allowing the whole brain to be imaged in a single bed position. The properties of this scanner have been reported elsewhere [26]. (*R*)-[^11^C]verapamil was synthesised as described previously [27]. Prior to tracer injection, a 10-min transmission scan in 2D acquisition mode was performed using three rotating ^68^Ge rod sources. This scan was used to correct the subsequent emission scan for photon attenuation. Next, a dynamic emission scan in 3D acquisition mode was started simultaneously with an intravenous injection of approximately 370 MBq (*R*)-[^11^C]verapamil. (*R*)-[^11^C]verapamil was injected at a rate of 0.8 mL·s^-1^, followed by a flush of 42 mL saline at 2.0 mL·s^-1 ^using an infusion pump (Med-Rad, Beek, The Netherlands). The emission scan consisted of 20 frames with a progressive increase in frame duration (1 × 15, 3 × 5, 3 × 10, 2 × 30, 3 × 60, 2 × 150, 2 × 300 and 4 × 600 s) and a total scan duration of 60 min. During the (*R*)-[^11^C]verapamil scan, arterial blood was withdrawn continuously using an automatic on-line blood sampler (Veenstra Instruments, Joure, The Netherlands [28]) at a rate of 5 mL·min^-1 ^for the first 5 min and 2.5 mL·min^-1 ^thereafter. At 2.5, 5, 10, 20, 30, 40 and 60 min after tracer injection, continuous blood sampling was interrupted briefly to withdraw a 10-mL manual blood sample, followed by flushing of the arterial line with a heparinised saline solution. These manual samples were used to determine plasma to whole blood [P/WB] radioactivity concentrations. In addition, concentrations of radioactive parent tracer and its polar metabolites in plasma were determined using a combination of solid-phase extraction and high-performance liquid chromatography, as described previously [29]. Patient movement was restricted by the use of a head holder and monitored by checking the position of the head using laser beams.

### PET data analysis

All PET data were corrected for attenuation, randoms, dead time, scatter and decay. Images were reconstructed using a standard filtered back projection [FBP] algorithm, applying a Hanning filter with a cutoff at 0.5 times the Nyquist frequency. A zoom factor of 2.123 and a matrix size of 256 × 256 × 63 were used, resulting in a voxel size of 1.2 × 1.2 × 2.4 mm and a spatial resolution of approximately 6.5 mm full width at half maximum at the centre of the field of view. Images were also reconstructed using a partial volume corrected ordered subset expectation maximization [PVC OSEM] reconstruction algorithm, a previously described and validated method that results in improved image resolution, thereby reducing partial volume effects [PVEs] [30-32]. Co-registration of structural T1 MRI images with corresponding summed FBP or PVC OSEM reconstructed (*R*)-[^11^C]verapamil images (frames 3 to 12) and segmentation of co-registered MRI images into grey matter, white matter and extracellular fluid was performed using statistical parametrical mapping (SPM, version SPM2, http://www.fil.ion.ucl.ac.uk/spm, Institute of Neurology, London, UK) software. ROIs were defined on the segmented MRI using a probabilistic template as implemented in the PVElab software [33]. The following ROIs were used for further analysis: frontal (volume-weighted average of orbital frontal, medial inferior frontal and superior frontal), parietal, temporal (volume-weighted average of superior temporal and medial inferior temporal), occipital, posterior and anterior cingulate, medial temporal lobe [MTL] (volume-weighted average of hippocampus and enthorinal) and cerebellum. In addition, a global cortical region was defined consisting of the volume-weighted average of frontal, parietal, temporal and occipital cortices and posterior and anterior cingulate regions. ROIs were mapped onto dynamic PET images, and regional time-activity curves were generated.

The on-line blood curve was calibrated using the seven manual whole blood samples. Next, the total plasma curve was obtained by multiplying this calibrated whole blood curve with a single-exponential function derived from the best fit to the P/WB ratios. Finally, the corrected plasma input function was generated by multiplying this total plasma curve with a sigmoid function derived from the best fit to one minus the polar fraction [19,34].

**Figure 1:**
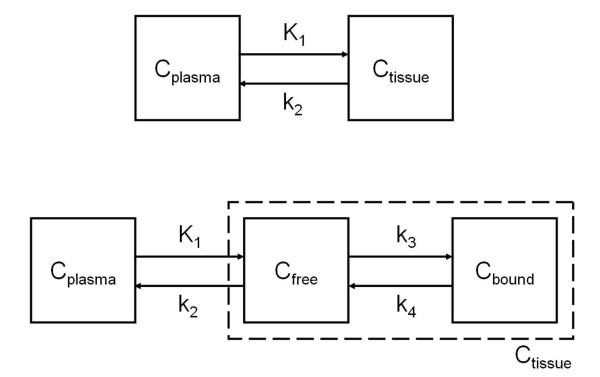
**Schematic diagrams of the compartment models**. In the upper diagram, a standard single-tissue compartment (1T2k) is shown. In this study, two different implementations were used: the 1T2k^60 ^model using 60 min of data acquisition and the 1T2k^10 ^model using the first 10 min of data acquisition. In the lower diagram a standard two-tissue compartment (2T4k) model is shown. In this study, two different implementations were used: the 2T4k model without and the 2T4k^VTnsfix ^model with fixation of *K*_1_/*k*_2 _to the whole brain grey matter value. C, compartment.

Kinetic analyses of (*R*)-[^11^C]verapamil data were performed using software developed within Matlab 7.04 (The Mathworks, Natick, MA, USA). Data were analysed using different compartment models, schematically shown in Figure 1, and for different outcome measures, which have been proposed in previous studies as methods for analysing (*R*)-[^11^C]verapamil data. First, (*R*)-[^11^C]verapamil data were analysed using non-linear regression to a standard single-tissue compartment model covering both the entire 60 min (1T2k^60^) and only the first 10 min (1T2k^10^) of data collection, yielding *K*_1_, *k*_2_, volume of distribution *V*_T _and the fractional blood volume *V*_B_. In addition, standard two-tissue compartment models without (2T4K) and with fixing *K*_1_/*k*_2 _to the mean whole brain grey matter value (2T4k^VTnsfix^) were tested, yielding, in addition to the individual rate constants *K*_1 _to *k*_4 _and *V*_B_, the outcome measures *V*_T _and non-displaceable binding potential BP_ND_. Goodness of fits for the various models was assessed by means of the Akaike information criterion [AIC] [35].

### Statistical analysis

*P *values for assessing differences in characteristics between test and retest scans were obtained using Students *t *tests. Test-retest variability was calculated as the absolute difference between test and retest scans divided by the mean of these two scans. Differences in TRT variability between FBP and PVC OSEM reconstructed data were assessed using paired *t *tests. Furthermore, the level of agreement between test and retest scans was assessed using Bland-Altman analysis [36]; the difference in values between both measurements was plotted against their mean. Data are presented as mean ± standard deviation, unless otherwise stated.

## Results

Thirteen test and retest scans were performed. There were no differences in injected dose (test 361 ± 29 MBq, retest 374 ± 24 MBq; *p *= 0.23) and specific activity (test 44 ± 13 GBq μmol^-1^, retest, 49 ± 16 GBq μmol^-1^; *p *= 0.41) of (*R*)-[^11^C]verapamil between test and retest scans.

Two data sets had to be excluded from further analysis due to incomplete blood data. In one retest scan, the polar and parent fractions of the last manual sample were missing due to technical problems. Another retest scan clearly had erroneous values for the polar fraction of the last two manual samples. For the 11 subjects included in the analyses, TRT variability for the parent fraction (mean parent fraction of samples 6 and 7 at 40 and 60 min, respectively) ranged from 2% to 26% in individual subjects, with a mean of 13 ± 8%.

**Figure 2:**
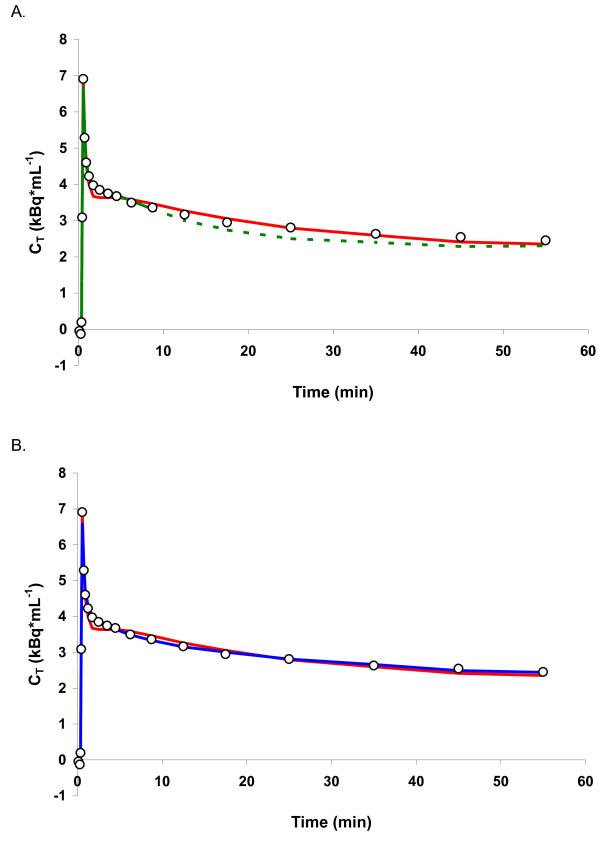
**Examples of various fits**. (**A**) The standard single-tissue compartment models fitted to the entire 60 min (1T2k^60^, red line) and only to the first 10 min (1T2k^10^, green line) of data collection. The dashed green line represents an extrapolation of the 1T2k^10 ^fit, i.e. data from 10 to 60 min were not used for fitting. (**B**) Fits obtained with the standard single-tissue compartment model (1T2k^60^, red line) and the two-tissue compartment model with fixed *K*_1_/*k*_2 _(2T4k^VTnsfix^, blue line). Fits of the unconstrained (standard) two-tissue compartment model (2T4k) were identical to those of the 2T4k^VTnsfix ^model.

First, fits to the various models for the global cortical region were assessed using AIC. The 1T2k^10 ^model was excluded from this analysis as it covers only 10 min rather than the entire 60 min of data acquisition. Since the 1T2k^10 ^model differs in the number of data points (fewer frames and shorter scan duration) from the other models, AIC values cannot be compared with the other models. For FBP reconstructed data, the 2T4k^VTnsfix ^model provided best fits in 19 out of 22 scans (86%) according to the AIC with a mean value of -98 ± 13. The 1T2k^60 ^and 2T4k models provided best fits in 1 (5%) and 2 (9%) out of 22 scans with mean AIC values of -81 ± 13 and -96 ± 14, respectively. Examples of the various model fits are shown in Figure 2. Similar results were obtained for PVC OSEM reconstructed PET data, with the lowest AIC (-103 ± 11) for the 2T4k^VTnsfix ^model in 17 out of 22 scans (77%). The 1T2k^60 ^model (mean AIC value -88 ± 13) and 2T4k model (mean AIC value -101 ± 11) provided best fits in 2 (9%) and 3 (14%) out of 22 scans, respectively.

Table 1 summarises TRT variability of the various outcome measures and parameters derived from FBP reconstructed (*R*)-[^11^C]verapamil data for all ROIs investigated. Average TRT variability of the 1T2k^60 ^model-derived *V*_T _for the global cortical brain region was 6.2%, and regional TRT variability ranged from 5.8% in the occipital to 8.3% in the posterior cingulate region. Corresponding TRT variabilities of the rate constants *K*_1 _and *k*_2 _for the global cortical region were 9.1 and 10.0%, respectively. Regional data are summarised in Table 2.

**Table 1:** Test-retest variability (%) of various outcome measures of (*R*)-[^11^C]verapamil kinetics derived from filtered back projection data

TRT (%)	1T2k^60^	1T2k^10^	2T4k^VTnsfix^	2T4k^VTnsfix^
	*V*_T_	*K*_1_	BP_ND_	*V*_T_
Global	6.2 ± 4.0	8.8 ± 6.4	22.0 ± 29.6	8.9 ± 6.8
Frontal	6.2 ± 3.9	9.1 ± 6.6	22.9 ± 27.8	9.6 ± 7.1
Parietal	6.0 ± 4.3	9.1 ± 5.5	22.9 ± 28.0	10.2 ± 7.5
Temporal	6.8 ± 4.1	8.6 ± 6.1	22.9 ± 29.7	7.9 ± 6.7
Occipital	5.8 ± 4.7	8.6 ± 7.6	22.5 ± 27.4	11.0 ± 7.4
Posterior cingulate	8.3 ± 6.0	11.1 ± 8.8	29.8 ± 37.0	13.6 ± 8.8
Anterior cingulate	7.0 ± 5.8	10.5 ± 5.7	27.6 ± 30.9	9.8 ± 7.4
Medial temporal	7.8 ± 5.0	12.7 ± 9.6	25.5 ± 25.0	11.5 ± 6.2
Cerebellum	6.8 ± 6.6	10.4 ± 7.8	25.3 ± 27.0	13.2 ± 11.2

**Table 2:** Test-retest variability (%) of various (*R*)-[^11^C]verapamil rate constants derived from filtered back projection reconstructed data

TRT (%)	1T2k^60^	1T2k^60^	1T2k^10^	1T2k^10^	2T4k^VTnsfix^	2T4k^VTnsfix^	2T4k^VTnsfix^	2T4k^VTnsfix^
	*K*_1_	*k*_2_	*V*_T_	*k*_2_	*K*_1_	*k*_2_	*k*_3_	*k*_4_
Global	9.1 ± 7.0	10.0 ± 6.0	5.9 ± 5.9	9.2 ± 5.1	9.1 ± 7.0	19.2 ± 27.1	66.2 ± 56.4	60.6 ± 45.0
Frontal	10.2 ± 6.7	10.3 ± 5.7	6.9 ± 6.3	9.2 ± 5.7	10.0 ± 6.4	19.6 ± 27.1	63.3 ± 56.1	58.5 ± 45.9
Parietal	9.4 ± 7.1	11.2 ± 6.0	6.9 ± 5.3	8.1 ± 7.1	9.2 ± 7.5	18.4 ± 27.0	63.1 ± 55.8	61.0 ± 45.3
Temporal	8.0 ± 6.4	8.9 ± 6.5	6.8 ± 5.3	11.3 ± 5.9	10.1 ± 6.3	20.1 ± 26.5	75.7 ± 57.3	65.7 ± 47.9
Occipital	9.7 ± 8.1	10.6 ± 5.0	6.6 ± 6.5	8.3 ± 4.9	8.3 ± 9.5	19.3 ± 28.8	68.8 ± 66.1	66.8 ± 59.1
Posterior cingulate	9.9 ± 10.3	9.5 ± 7.8	14.1 ± 14.4	16.8 ± 14.1	9.8 ± 8.3	21.1 ± 25.8	77.9 ± 65.4	73.7 ± 55.1
Anterior cingulate	9.7 ± 6.7	11.6 ± 6.6	16.7 ± 15.7	20.7 ± 18.1	10.2 ± 6.3	17.8 ± 25.5	71.0 ± 65.9	71.6 ± 54.5
Medial temporal	10.6 ± 9.3	11.1 ± 9.5	16.8 ± 12.6	25.1 ± 15.3	13.0 ± 8.1	22.7 ± 27.6	69.7 ± 39.5	60.6 ± 40.3
Cerebellum	10.9 ± 7.6	10.3 ± 6.7	6.8 ± 4.8	7.2 ± 5.7	10.2 ± 7.9	18.6 ± 27.0	58.1 ± 55.6	61.4 ± 44.1

For the 1T2k^10 ^model, TRT variability of the outcome measure *K*_1 _was 8.8% for the global cortical ROI and varied from 8.6% in both temporal and occipital regions to 12.7% in the medial temporal lobe region (Table 1). Corresponding TRT values for *V*_T _and *k*_2 _are listed in Table 2.

The standard 2T4k model resulted in outcome measures and rate constants that could not be determined reliably (i.e. very high standard errors [SEs] of fitted parameters). Therefore, assessment of TRT variability did not seem useful. SEs of outcome parameters from the other models were very acceptable. For example, for the global cortical region and FBP reconstructed data, SE values were in the range of 0.14% for *V*_T _(1T2k^60^), 2.7% for *K*_1 _(1T2k^10^), 3.3% for *V*_T _(2T4k^VTnsfix^) and 3.2% for BP_ND _(2T4k^VTnsfix^).

For the 2T4k^VTnsfix ^model, TRT variability of the outcome measure BP_ND _for the global cortical brain region was 22.0%, and regional TRT values varied from 22.5% in the occipital to 29.8% in the posterior cingulate region (Table 1). Corresponding TRT variability of *V*_T _for the global cortical region was 8.9% (Table 1). TRT values of the rate constants *K*_1 _to *k*_4 _for the 2T4k^VTnsfix ^model are given in Table 2.

**Figure 3:**
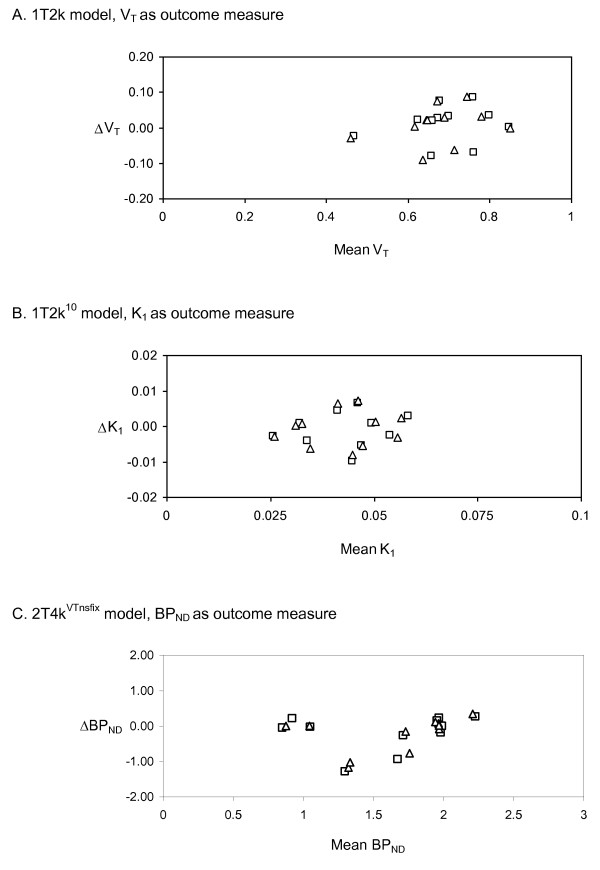
**Bland-Altman plots for the various outcome measures derived from FBP and PVC OSEM reconstructed data**. (**A**) 1T2k model, *V*_T _as outcome measure. (**B**) 1T2k^10 ^model, *K*_1 _as outcome measure. (**C**) 2T4k^VTnsfix ^model, BP_ND _as outcome measure. The Greek letter delta represents the change between test and retest values in the global cortical region. On the *x*-axis, the mean of test and retest values is given. Squares, FBP data; triangles, PVC OSEM data.

Tables 3 and 4 provide similar data as Tables 1 and 2, but now for PVC OSEM rather than FBP reconstructed data. Although there was some regional variation, TRT variability of all parameters derived from all models was comparable, though not exactly the same as for FBP reconstructed data. Although TRT variabilities of *K*_1 _obtained with the 1T2k^10 ^model and BP_ND _and *V*_T _obtained with the 2T4k^VTnsfix ^model were slightly higher for PVC OSEM reconstructed data, these differences between both reconstruction methods were not statistically significant (tested using paired *t *tests) for any of the regions assessed. Next, the level of agreement between test and retest scans was assessed by plotting the difference in values between both measurements against their mean for the various outcome measures, as shown in Figure 3.

**Table 3:** Test-retest variability (%) of various outcome measures of (*R*)-[^11^C]verapamil kinetics derived from PVC OSEM reconstructed data

TRT (%)	1T2k^60^	1T2k^10^	2T4k^VTnsfix^	2T4k^VTnsfix^
	*V*_T_	*K*_1_	BP_ND_	*V*_T_
Global	6.3 ± 4.7	9.6 ± 6.7	22.7 ± 32.2	9.0 ± 6.2
Frontal	6.4 ± 4.8	9.2 ± 6.2	24.7 ± 30.0	9.0 ± 7.1
Parietal	5.7 ± 3.7	10.6 ± 7.1	23.3 ± 31.0	9.4 ± 5.7
Temporal	7.2 ± 4.9	9.3 ± 6.6	25.8 ± 30.9	9.2 ± 6.4
Occipital	6.8 ± 6.1	10.8 ± 7.4	23.2 ± 32.1	10.0 ± 7.2
Posterior cingulate	9.3 ± 6.9	13.3 ± 10.1	33.5 ± 37.4	13.1 ± 8.8
Anterior cingulate	5.9 ± 5.2	14.2 ± 5.8	28.5 ± 34.2	8.5 ± 5.4
Medial temporal	11.8 ± 10.8	18.9 ± 23.1	38.8 ± 32.5	18.6 ± 19.9
Cerebellum	6.3 ± 4.5	7.6 ± 5.6	26.2 ± 30.9	10.6 ± 6.1

**Table 4:** Test-retest variability (%) of various (*R*)-[^11^C]verapamil rate constants derived from PVC OSEM reconstructed data

TRT (%)	1T2k^60^	1T2k^60^	1T2k^10^	1T2k^10^	2T4k^VTnsfix^	2T4k^VTnsfix^	2T4k^VTnsfix^	2T4k^VTnsfix^
	*K*_1_	*k*_2_	*V*_T_	*k*_2_	*K*_1_	*k*_2_	*k*_3_	*k*_4_
Global	9.9 ± 8.0	10.2 ± 7.2	7.4 ± 7.3	9.7 ± 6.3	8.2 ± 6.5	21.9 ± 26.1	62.2 ± 54.4	50.2 ± 38.2
Frontal	10.1 ± 7.9	10.4 ± 8.0	9.3 ± 9.2	11.1 ± 7.7	7.6 ± 5.1	21.3 ± 25.1	61.5 ± 57.8	51.0 ± 43.6
Parietal	10.7 ± 8.1	11.3 ± 6.9	9.0 ± 5.9	11.5 ± 9.9	9.7 ± 7.8	22.9 ± 26.0	66.1 ± 57.5	57.7 ± 40.2
Temporal	9.1 ± 8.2	11.6 ± 6.8	7.1 ± 5.8	9.4 ± 6.2	8.0 ± 7.2	22.2 ± 26.1	61.7 ± 52.6	49.3 ± 36.1
Occipital	11.0 ± 7.6	9.3 ± 7.1	6.7 ± 7.5	9.7 ± 6.7	10.7 ± 7.5	23.1 ± 28.8	60.0 ± 56.1	49.1 ± 39.1
Posterior cingulate	13.4 ± 11.5	11.4 ± 8.2	15.6 ± 10.1	17.7 ± 9.5	13.6 ± 10.6	28.0 ± 27.4	84.4 ± 57.1	69.8 ± 54.1
Anterior cingulate	12.9 ± 9.3	12.6 ± 8.8	13.8 ± 8.6	21.7 ± 12.5	11.3 ± 6.2	23.3 ± 24.6	74.7 ± 63.8	65.4 ± 50.7
Medial temporal	15.0 ± 21.7	14.4 ± 13.0	25.8 ± 13.2	38.3 ± 22.1	16.9 ± 19.1	28.6 ± 31.1	82.3 ± 53.5	79.2 ± 45.5
Cerebellum	8.4 ± 7.5	10.2 ± 6.8	10.1 ± 6.4	10.6 ± 6.7	7.2 ± 5.9	20.8 ± 26.1	68.0 ± 60.8	59.1 ± 47.5

**Figure 4:**
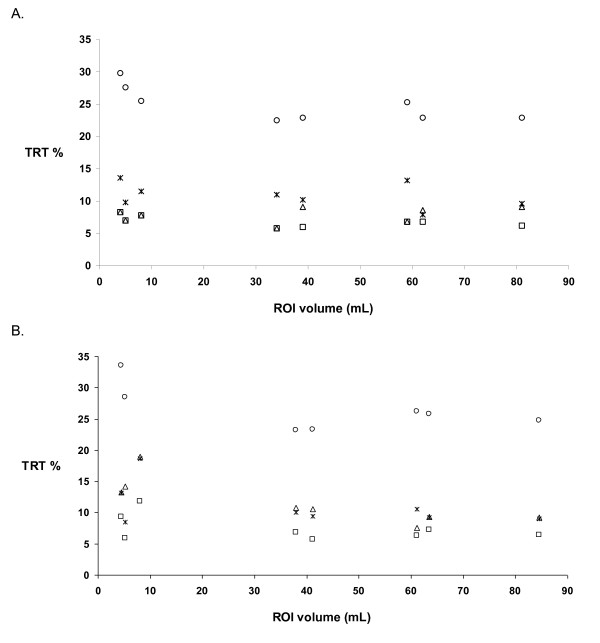
**Test-retest variability (TRT %) as a function of ROI volume**. (**A**) FBP reconstructed data and (**B**) PVC OSEM reconstructed data. Squares, 1T2k^60 ^model with outcome measure *V*_T_; triangles, 1T2k^10 ^model with outcome measure *K*_1_; circles, 2T4k^VTnsfix ^model with outcome measure BP_ND_; crosses, 2T4k^VTnsfix ^model with outcome measure *V*_T_.

The global cortical brain region was the largest brain region assessed with a mean volume of 226 ± 29 mL. Apart from the global cortical region, which consists of six smaller brain regions, the frontal region was the largest region with a mean volume of 81 ± 8 mL, whereas the posterior cingulate was the smallest with a mean volume of 4 ± 1 mL. Figure 4 shows TRT variability as a function of the mean ROI size for FBP reconstructed data (Figure 4A) and for PVC OSEM reconstructed data (Figure 4B).

## Discussion

This study evaluated test-retest variability of (*R*)-[^11^C]verapamil data using several tracer kinetic models. Of the three outcome measures that have been suggested to reflect Pgp function, the best TRT variability was found for *V*_T _using the 1T2k^60 ^model (global TRT 6%). Using the 2T4k^VTnsfix ^model, comparable TRT variability was found for *V*_T _(global TRT 9%), but TRT variability for BP_ND _was higher (global TRT 22%). For *K*_1 _derived from the 1T2k^10 ^model, global TRT variability was 9%. TRT variability could not be assessed for the 2T4k model without fixing *K*_1_/*k*_2 _to a global value. In a previous study evaluating several compartment models for (*R*)-[^11^C]verapamil data, it has also been shown that TRT variability was substantially higher for a 2T4k model, and in that study, it was concluded that the 1T2k model was the model of choice for analysing (*R*)-[^11^C]verapamil data [19]. Nevertheless, in this study, AIC analysis showed that the 2T4k^VTnsfix ^model provided better fits to the data than the standard single-tissue compartment model, with substantial differences in AIC values. Furthermore, test-retest variability and precision of the fitted outcome measures were very acceptable. Regarding the 1T2k^10 ^model as proposed by Muzi et al., TRT variability of the outcome measure *K*_1 _was moderate; the quality of the fit (over the first 10 min) was good, and a shorter scan duration is an advantage, especially in certain patient groups. Nevertheless, *K*_1 _might not fully reflect Pgp function. Although a significant increase in *K*_1 _was found after Pgp inhibition, there was an even larger increase in *k*_3 _[23]. In addition, previous studies as well as spectral analysis have shown that there are two compartments in (*R*)-[^11^C]verapamil data, in healthy controls under baseline conditions, in Alzheimer's disease patients [9] and especially after pharmacological blockade of Pgp [21,23]. Therefore, despite its slightly higher TRT of *V*_T_, the 2T4k^VTnsfix ^model is the tracer kinetic model of choice, even for baseline studies in healthy controls. Although TRT variability of BP_ND _was higher, TRT variability of *V*_T _was quite similar for the constrained two-tissue and standard single-tissue compartment models. Therefore, *V*_T _derived from the constrained two-tissue compartment model should be used. This has the further advantage that the same model can be used in blocking experiments, where baseline scans are compared with scans after administration of a Pgp inhibitor, or when comparing different groups of patients.

The present study is the first to assess TRT variability of regional (*R*)-[^11^C]verapamil data as previous studies have reported on total brain TRT variability only [19]. Although there is a slight decrease (approximately 5%) in reproducibility for brain regions with the smallest volumes, such as the anterior and posterior cingulate, this effect is only marginal (Figure 4). The slightly higher TRT values in the medial temporal lobe (Tables 1 and 3) may be secondary to spill over from the very high signal in the choroid plexus.

The effect of PVE correction methods on TRT variability of (*R*)-[^11^C]verapamil data has not been assessed before. In the present study, images were reconstructed using both standard FBP and PVC OSEM reconstruction algorithms [30]. PVC OSEM improves in-plane resolution of PET images by taking the point spread function of the scanner into account, leading to reduced PVEs [31]. Interestingly, differences in TRT variability between PVC OSEM and FBP reconstructed data were only minor (Tables 1 and 3). It should, however, be noted that only healthy controls were included, and although the age range varied from 21 to 63 years, there was no significant brain atrophy present on MRI scans. The effects of PVE correction methods and their impact on TRT variability should be assessed in future studies in conditions where brain atrophy may be present, such as in neurodegenerative diseases. However, as (*R*)-[^11^C]verapamil is a tracer which has low uptake throughout the brain and therefore shows little contrast, no major effects from PVE correction methods should be expected. Even in the medial temporal lobe, where the signal was higher than in other brain regions, no improvement in TRT variability was seen. In fact, TRT variability in this region was higher after PVE correction. For MTL, PVE correction implies a small signal following a large correction for PVEs. Consequently, noise levels in the corrected MTL signal will be higher than in other regions, resulting in higher TRT values.

In conclusion, reproducibility of (*R*)-[^11^C]verapamil PET studies was best for *V*_T _derived from single-tissue (6%) and constrained two-tissue (9%) compartment models. As the constrained two-tissue compartment model provided the best fits to the data, it is the kinetic model of choice with the volume of distribution *V*_T _as the preferred outcome measure.

## Competing interests

The authors declare that they have no competing interests.

## Authors' contributions

DMEvA performed the PET studies and data analysis and wrote the manuscript, ML was involved in the model development and data processing. RB was involved in the quality control of PET data. RCS performed the metabolite analysis and quality control of the tracer. ADW was involved in the tracer production and quality control of tracer production processes. PS helped in drafting the manuscript. AAL was involved in the study design and helped in drafting the manuscript. BNMvB supervised the PET data acquisition and helped in drafting the manuscript. All authors have read and approved the final manuscript.
